# Correction: *Mycobacterium tuberculosis* PhoP integrates stress response to intracellular survival by regulating cAMP level

**DOI:** 10.7554/eLife.105750

**Published:** 2024-12-20

**Authors:** Hina Khan, Partha Paul, Harsh Goar, Bhanwar Bamnia, Navin Baid, Dibyendu Sarkar

**Keywords:** Other

Khan H, Paul P, Goar H, Bamniya B, Baid N, Sarkar D. 2024. Mycobacterium tuberculosis PhoP integrates stress response to intracellular survival by regulating cAMP level. *eLife* **13**:RP92136. doi: 10.7554/eLife.92136.

Published 13 May 2024

It has come to our attention that a reader recently pointed out errors in the statistics of our published article, where the significance levels were inconsistent with the underlying source data provided. These errors in Figures 1B, 2A, 2B, 3C, 3D, 4B and Figure 3-figure supplement 1 were caused during the assembly of the final Excel files for publication. Additionally, we identified several other errors that require correction. This includes an error in Figure 3D due to the incorrect calculation of standard error of *rv0805* expression in *phoP*-KD mutant [from 0.967 (original standard error) to 0.319 (corrected standard error)]. Errors in Figures 4A and 4C, where replicates of a few datasets were unintentionally omitted from the calculations, and one data set was utilized in plotting instead of using the average of three datasets. Minor corrections to the wording of two sentences within the Results section were incorporated for better clarity. None of the outcomes or conclusions of the manuscript are affected by the corrections. We sincerely apologise for the oversight.

This necessitated the changes to the Results, Materials and Methods, and Figure and legends as described below. Underline indicates differences between corrected and original text.

## Results: Intra-mycobacterial cAMP level is regulated by the *phoP* locus

Corrected text: #1

Complementation of the mutant (Compl.) could restore cAMP levels. Under normal conditions and NO stress, a higher cAMP level in the complemented strain is possibly attributable to reproducibly higher *phoP* expression in the complemented mutant (Khan et al., 2022).

Original text: #1

Complementation of the mutant (Compl.) could restore cAMP to the WT level. A higher cAMP level in the complemented strain under NO stress is possibly attributable to reproducibly higher *phoP* expression in the complemented mutant under specific stress conditions (Khan et al., 2022).

Corrected text: #2

From these results, we conclude that PhoP is controlling cAMP level mainly in stressed cells.

Original text: #2

From these results, we conclude that PhoP plays a major role in maintaining intra-mycobacterial cAMP level.

## Materials and methods: Statistical analysis

We have now defined the significance levels once within the ‘Materials and methods’ section of the manuscript rather than repeating this for each figure legend.

Corrected text: #1

Data are presented as arithmetic means of the results obtained from multiple

replicate experiments ± standard deviations. Statistical significance was determined by Student’s paired t-test using Microsoft Excel or Graph Pad Prism. Statistical significance was considered at P values of 0.05 or lower (*p≤0.05; **p≤0.01; ***p≤0.001; ****p≤0.0001).

Original text: #1

Data are presented as arithmetic means of the results obtained from multiple

replicate experiments ± standard deviations. Statistical significance was determined by Student’s paired t-test using Microsoft Excel or Graph Pad Prism. Statistical significance was considered at P values of 0.05.

### Figures and legends

1) Figure 1: There was an unintentional error in significance (p-value) when comparing cAMP secretion by WT-H37Rv and *phoPR*-KO shown in Figure 1B. The two star significance (**) is now changed to non-significant (ns). The change had no net effect on the reported values. The error was corrected in the associated Figure.

Corrected text in Figure 1 legend:

‘….in the corresponding culture filtrates (CF) (ns., non- significant)...’

Original text in Figure 1 legend is shown for reference:

‘….in the corresponding culture filtrates (CF) …..’

2) Figure 2: There were unintentional errors in significance (p-values) when comparing *rv0891c* expression between WT and *phoPR*-KO shown in Figure 2A and *rv0805* expression in WT-H37Rv grown under pH 7.0 versus acidic pH (pH 4.5) shown in Figure 2B. In both Figures 2A and 2B, the two star (**) significance is now changed to one star (*) significance. These changes had no net effects on the reported values, but has a minor effect on statistics. The errors were corrected in the associated Figure.

Corrected text in Figure 2A legend:

‘….each with two technical repeats (*p<0.05; **p<0.01)…..’

Original text in Figure 2A legend is shown for reference:

‘….each with two technical repeats…..’

Corrected text in Figure 2B legend:

‘..as described in Materials and methods (*p<0.05)...’

Original text in Figure 2B legend is shown for reference:

‘..as described in Materials and methods (*p<0.01)...’

3) Figure 3: There have been errors in significance (p-values) while comparing cAMP levels of WT-Rv0805 and WT-Rv0805M in Figure 3C and *phoP* expression levels in WT and *phoP*-KD mutant in Figure 3D. Additionally, we spotted a minor error in the calculation of standard error of *rv0805* expression [0.319 (corrected) from the original (0.967)] of *phoP*-KD mutant reported in Figure 3D. The associated figure, legend, and source data file are corrected. The changes are shown in bold. We have now included corrected source data file of Figure 3D.

Corrected text in Figure 3C legend:

‘….Significance in variation of cAMP levels was determined by paired Student’s t-test (*p<0.05; **p<0.01)……..’

Original text in Figure 3C legend is shown for reference:

‘….Significance in variation of cAMP levels was determined by paired Student’s t-test (**p<0.01)…..’

4) Figure 4: In the source data file of Figure 4A, the significance (p-values) was unintentionally omitted. In Figure 4B, there has been an error in the significance (p-value) between WT and the *phoPR*-KO mutant. Additionally, in Figures 4A and 4C one set of the triplicate datasets were used in plotting. The associated figure, legend and source data file are corrected. The changes are shown in bold. The three data sets were only present in the source data file, and did not affect Figure 4. We have now included corrected source data files of Figures 4A and 4C.

Corrected text in Figure 4B legend:

‘…., was determined by plotting fluorescence intensity (*p<0.05; **p<0.01; ***p<0.001)....’

Original text in Figure 4B legend is shown for reference:

‘…., was determined by plotting fluorescence intensity….’

5) Figure 3-figure supplement 1-source data 1: There were unintentional errors in significance (p-values) when comparing *rv0805* expression between WT and WT-Rv0805 and between WT and WT-Rv0805M, respectively shown in Figure 3-figure supplement 1. In these cases, the three star (***) significance is now changed to two-star (**) significance and two-star significance (**) is now changed to one star (*) significance, respectively. These changes had no net effects on the reported values, but has a minor effect on statistics. The errors are corrected in the associated Figure.

Corrected text in Figure 3-figure supplement 1-source data 1 legend:

‘……as described in the Materials and methods (*p<0.05; **p<0.01)….....’

Original text in Figure 3-figure supplement 1-source data 1 legend is shown for reference:

‘……as described in the Materials and methods (**p≤0.01; ***p≤0.001)....’

The corrected Figure 1 is shown here:

**Figure 1:**
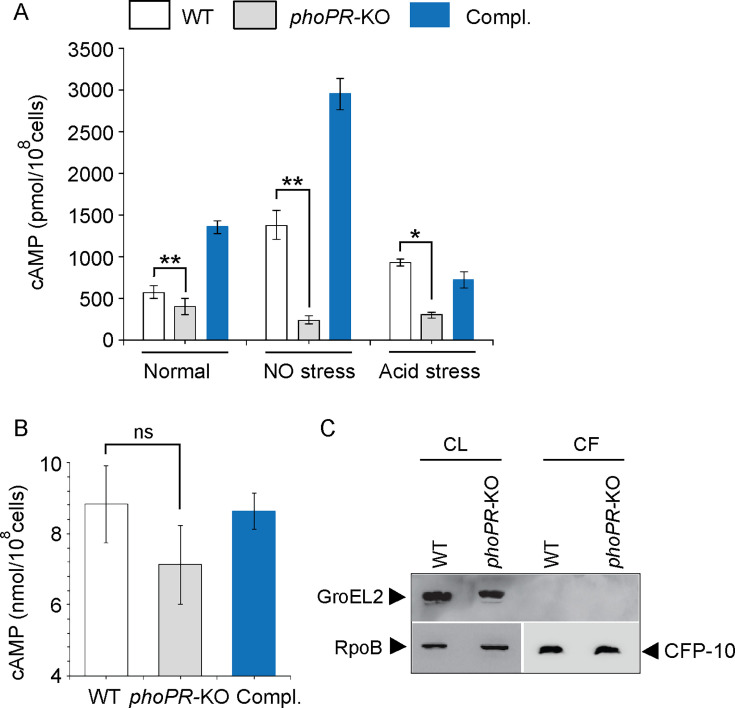


The originally published Figure 1 is shown for reference:

**Figure 2:**
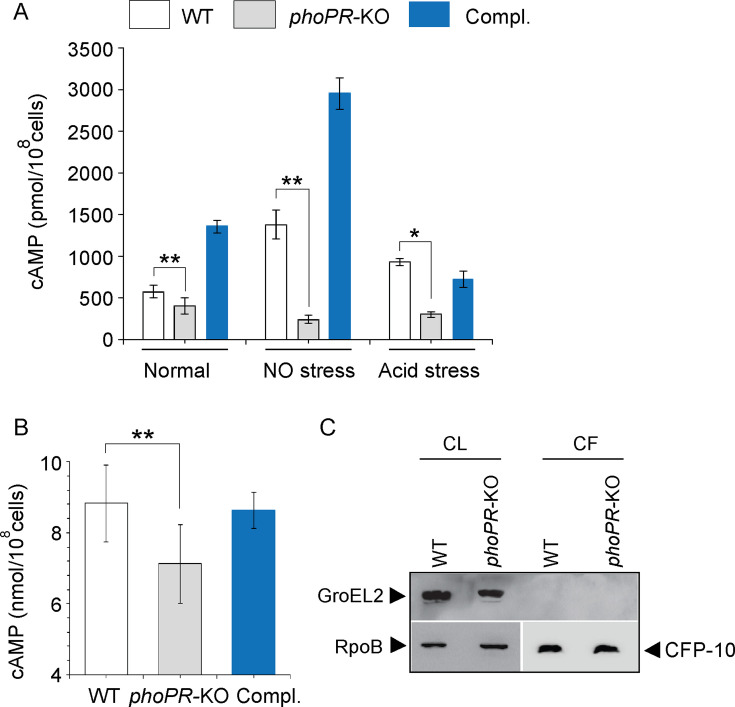


The corrected Figure 2 is shown here:

**Figure 3:**
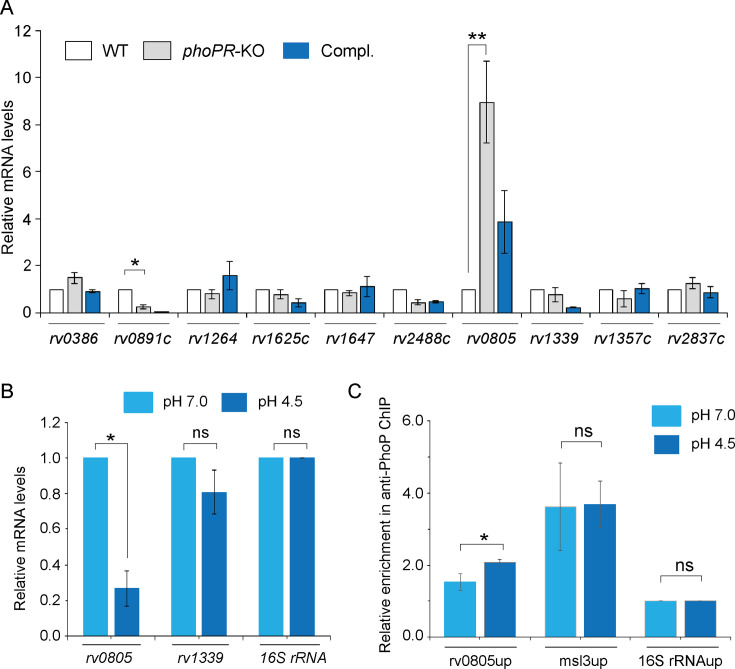


The originally published Figure 2 is shown for reference:

**Figure 4:**
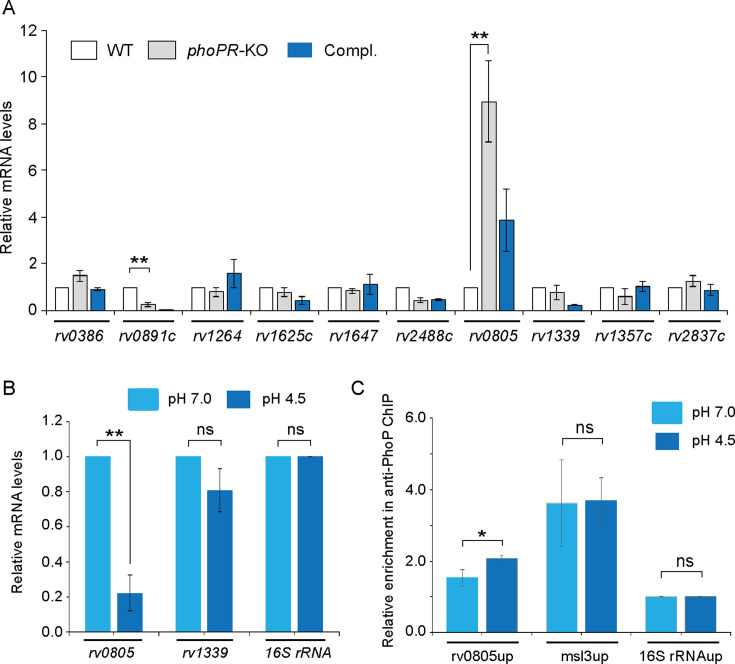


The corrected Figure 3 is shown here:

**Figure 5:**
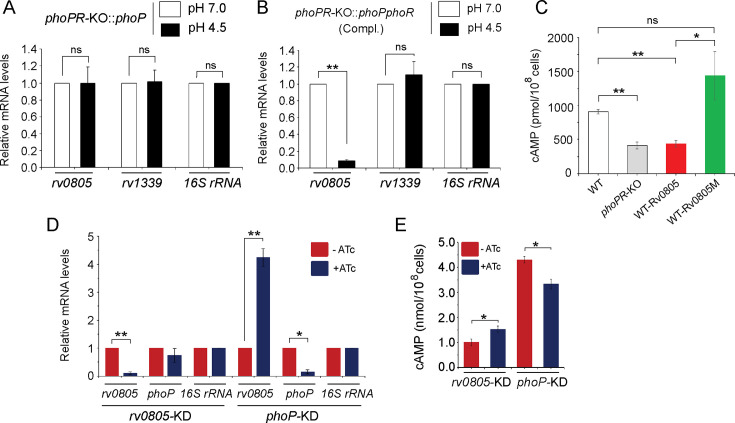


The originally published Figure 3 is shown for reference:

**Figure 6:**
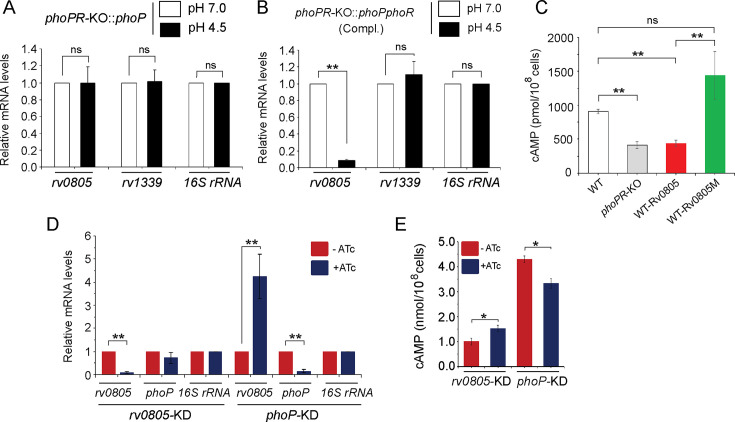


The corrected Figure 4 is shown here:

**Figure 7:**
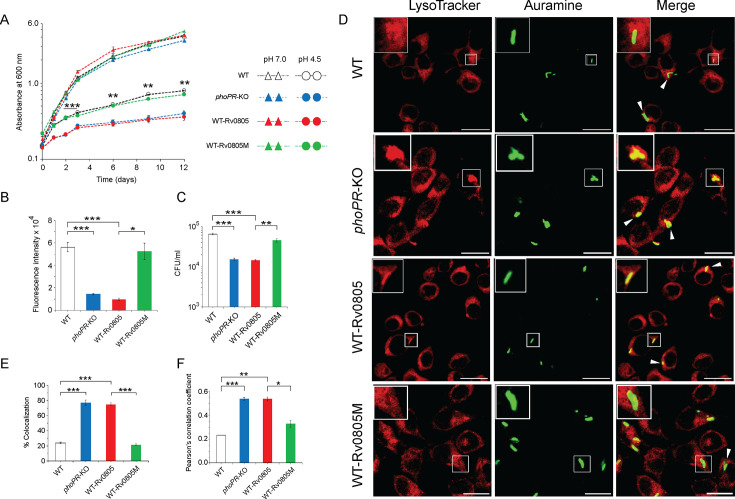


The originally published Figure 4 is shown for reference:

**Figure 8:**
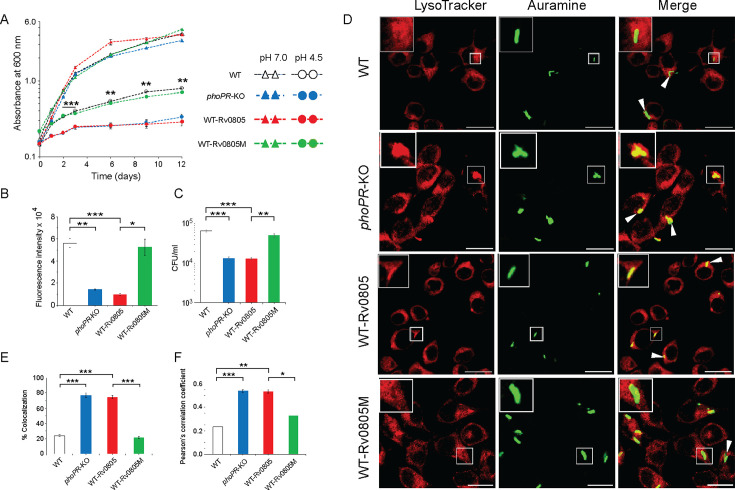


The article has been corrected accordingly.

