# Supplementary figures and images for: Mycobacterium tuberculosis PhoP integrates stress response to intracellular survival by regulating cAMP level
Source: eLife. 2024 May 13;13:RP92136. doi: 10.7554/eLife.92136 (PMC11090507; doi:10.7554/eLife.92136)

## Slide 1
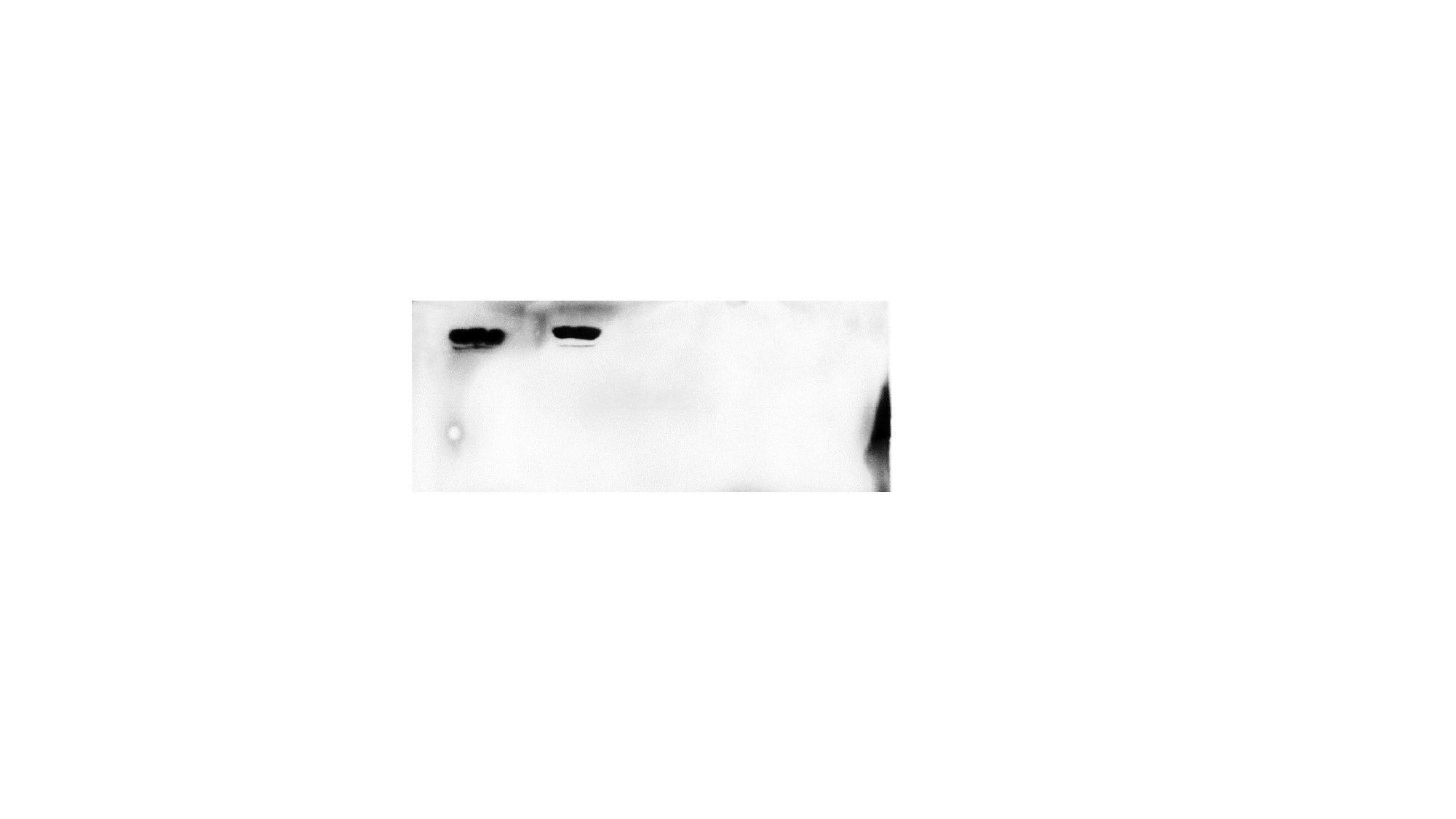

Supplement: Figure 1—source data 3. [file elife-92136-fig1-data3.pptx]

## Slide 1
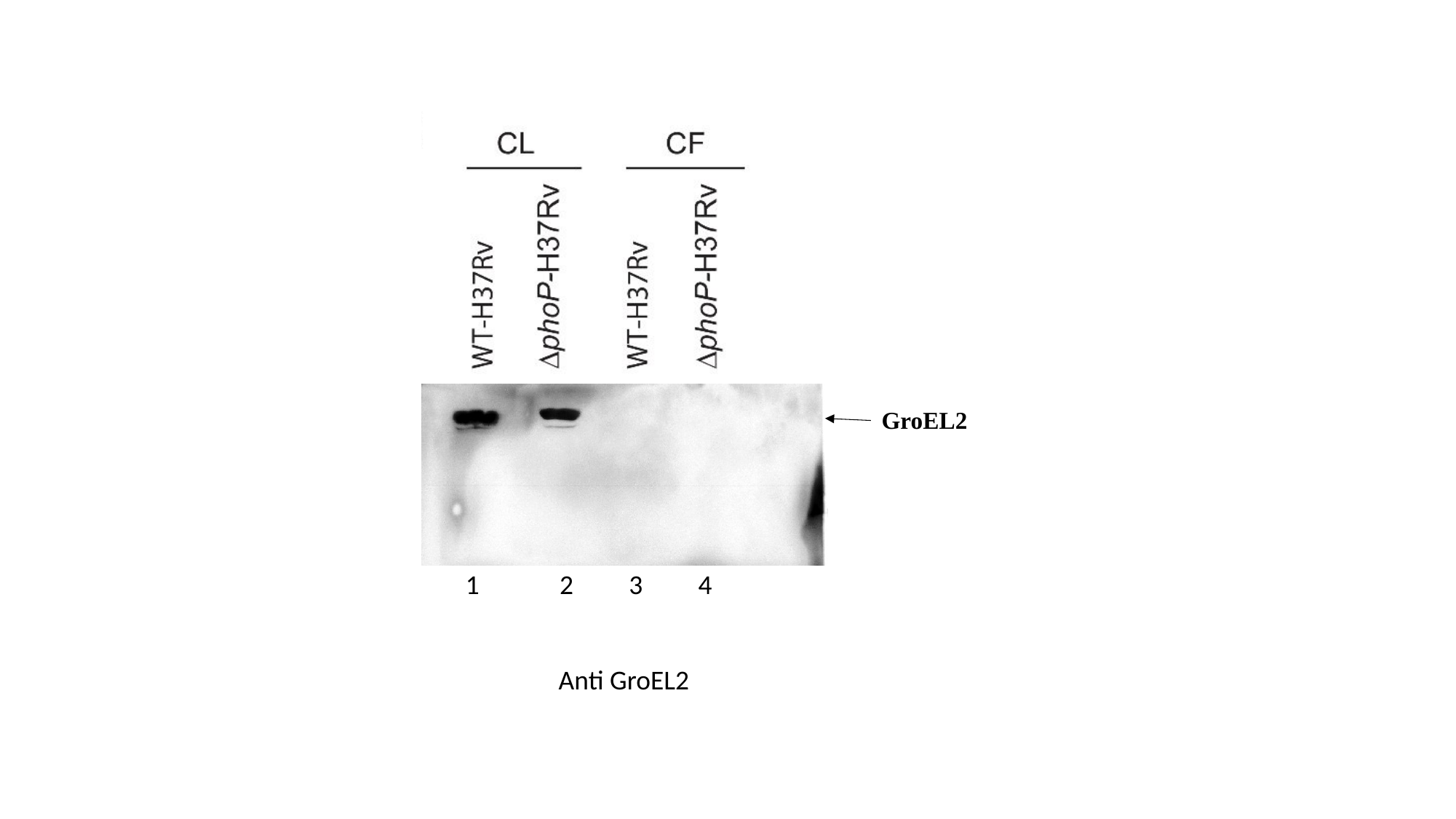

GroEL2
1 2 3 4
Anti GroEL2

Supplement: Figure 1—source data 4. [file elife-92136-fig1-data4.pptx]

## Slide 1
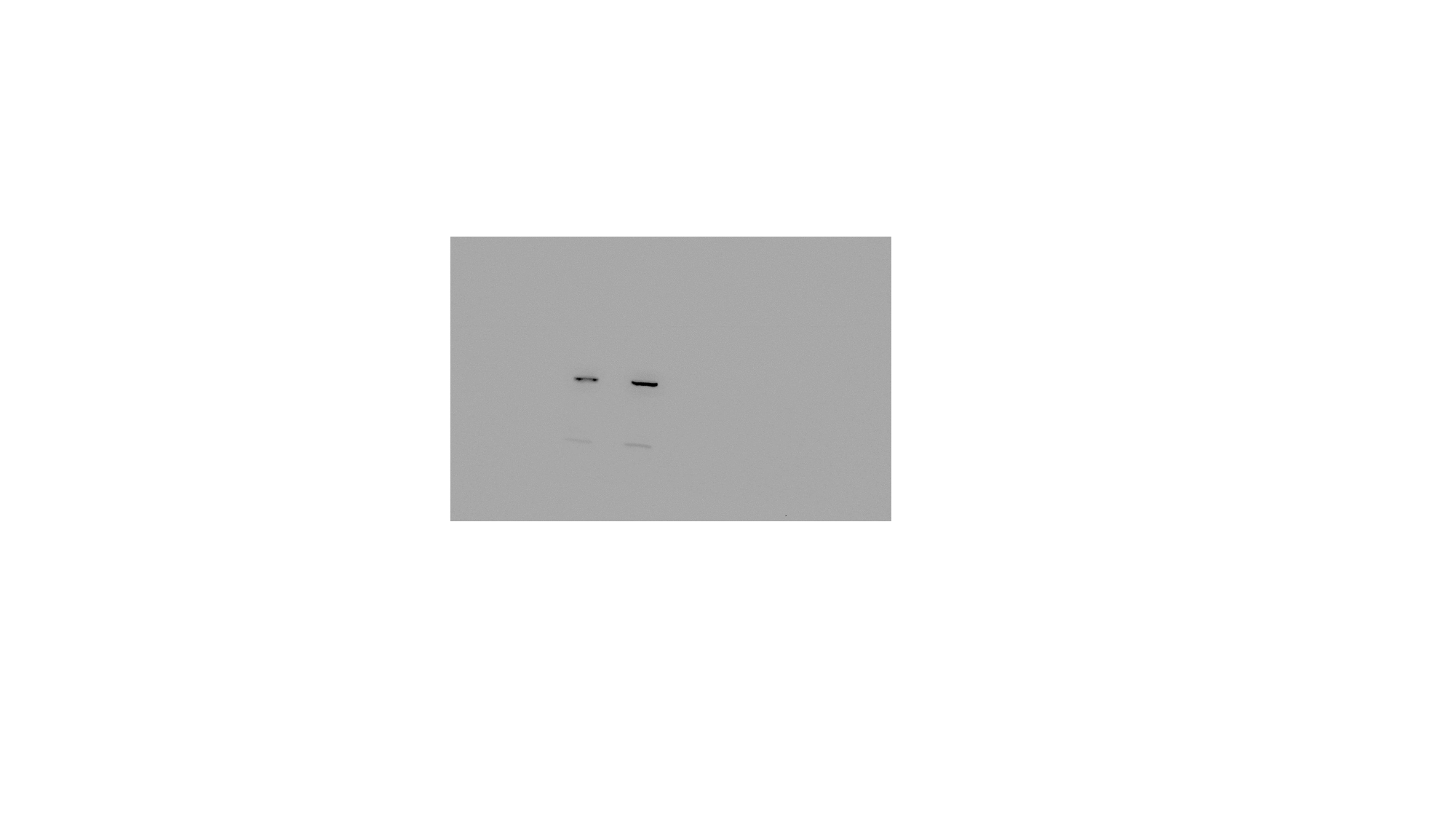

Supplement: Figure 1—source data 5. [file elife-92136-fig1-data5.pptx]

## Slide 1
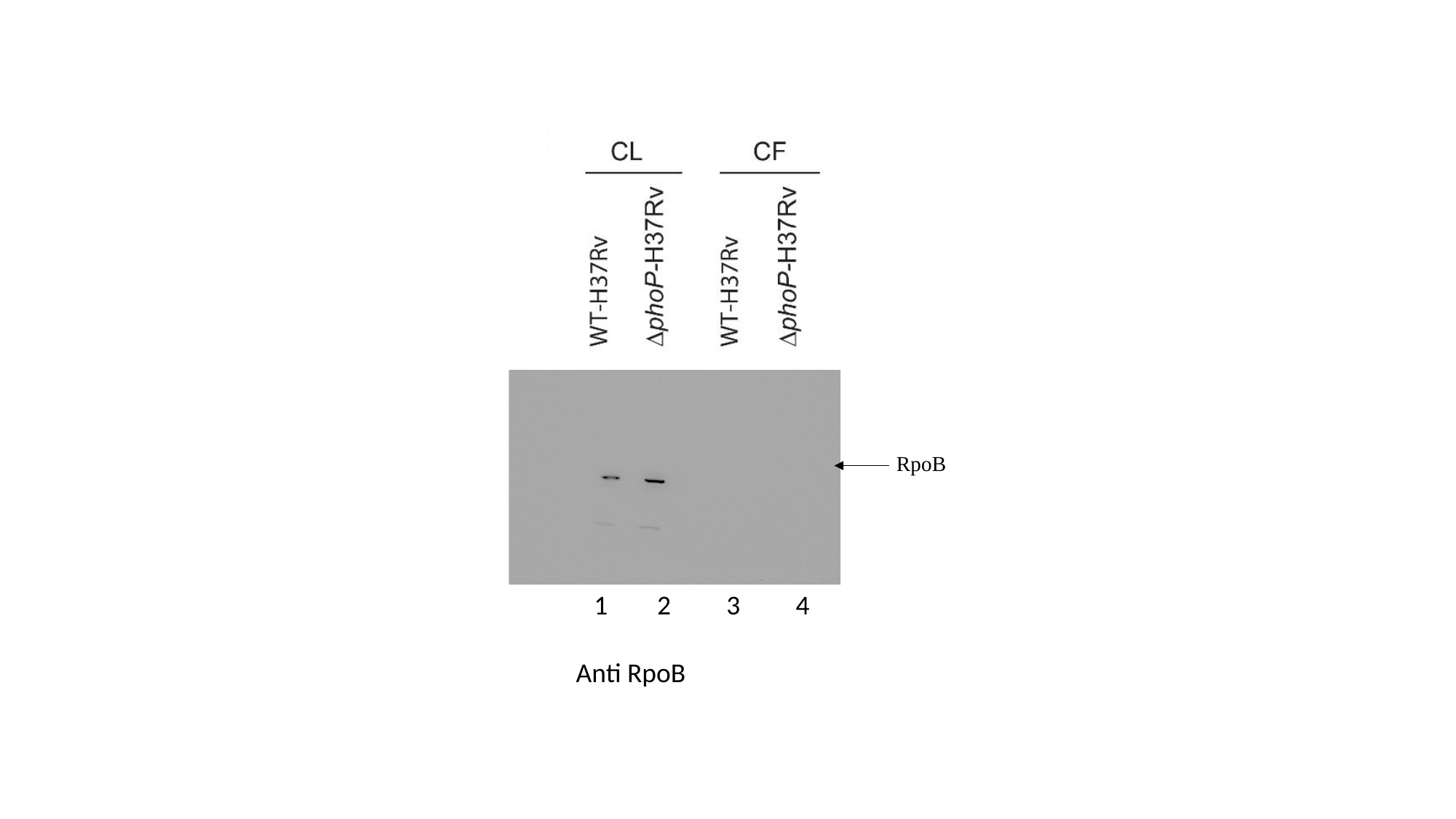

RpoB
1 2 3 4
Anti RpoB

Supplement: Figure 1—source data 6. [file elife-92136-fig1-data6.pptx]

## Slide 1
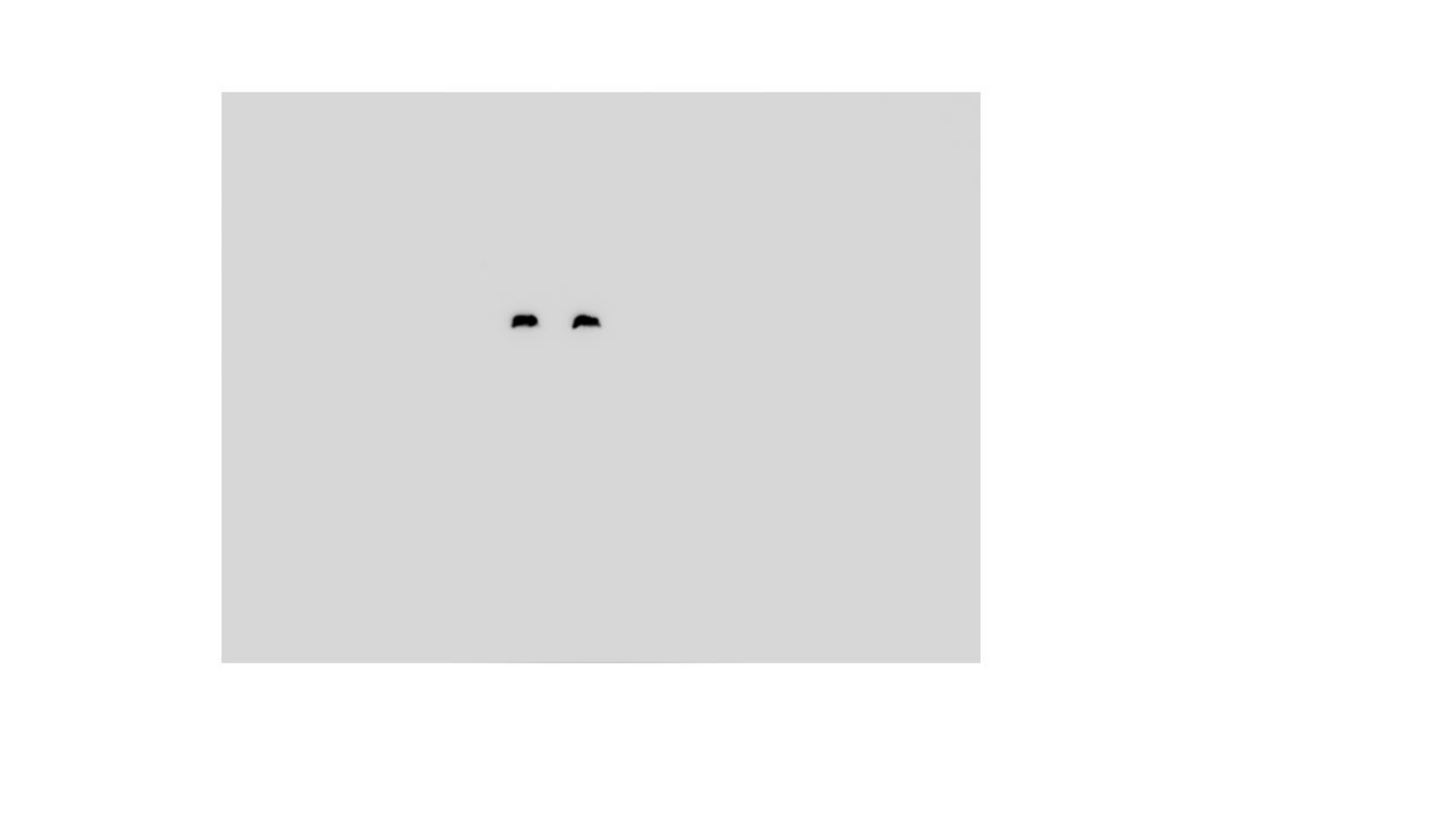

Supplement: Figure 1—source data 7. [file elife-92136-fig1-data7.pptx]

## Slide 1
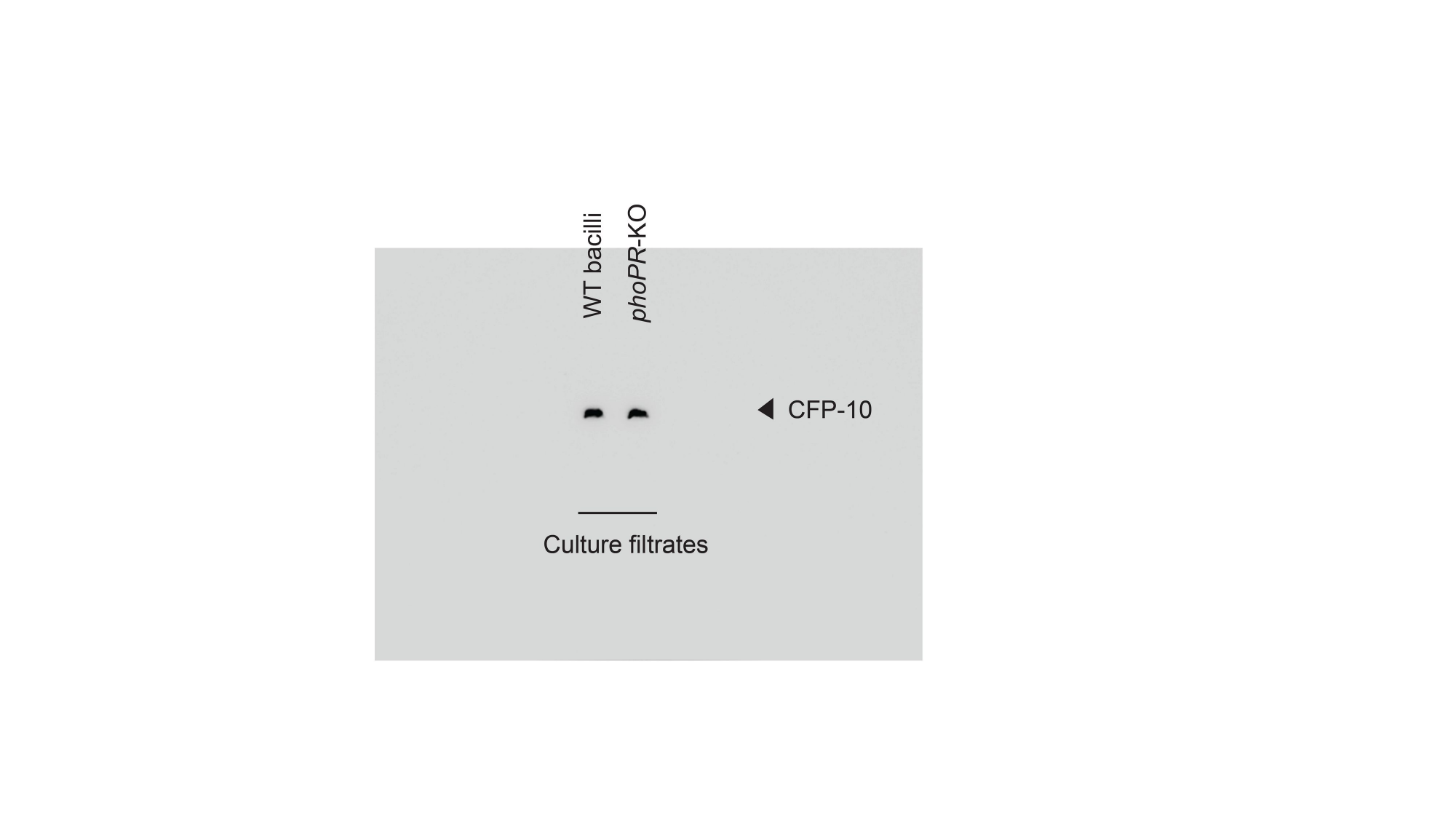

Supplement: Figure 1—source data 8. [file elife-92136-fig1-data8.pptx]

## Slide 1
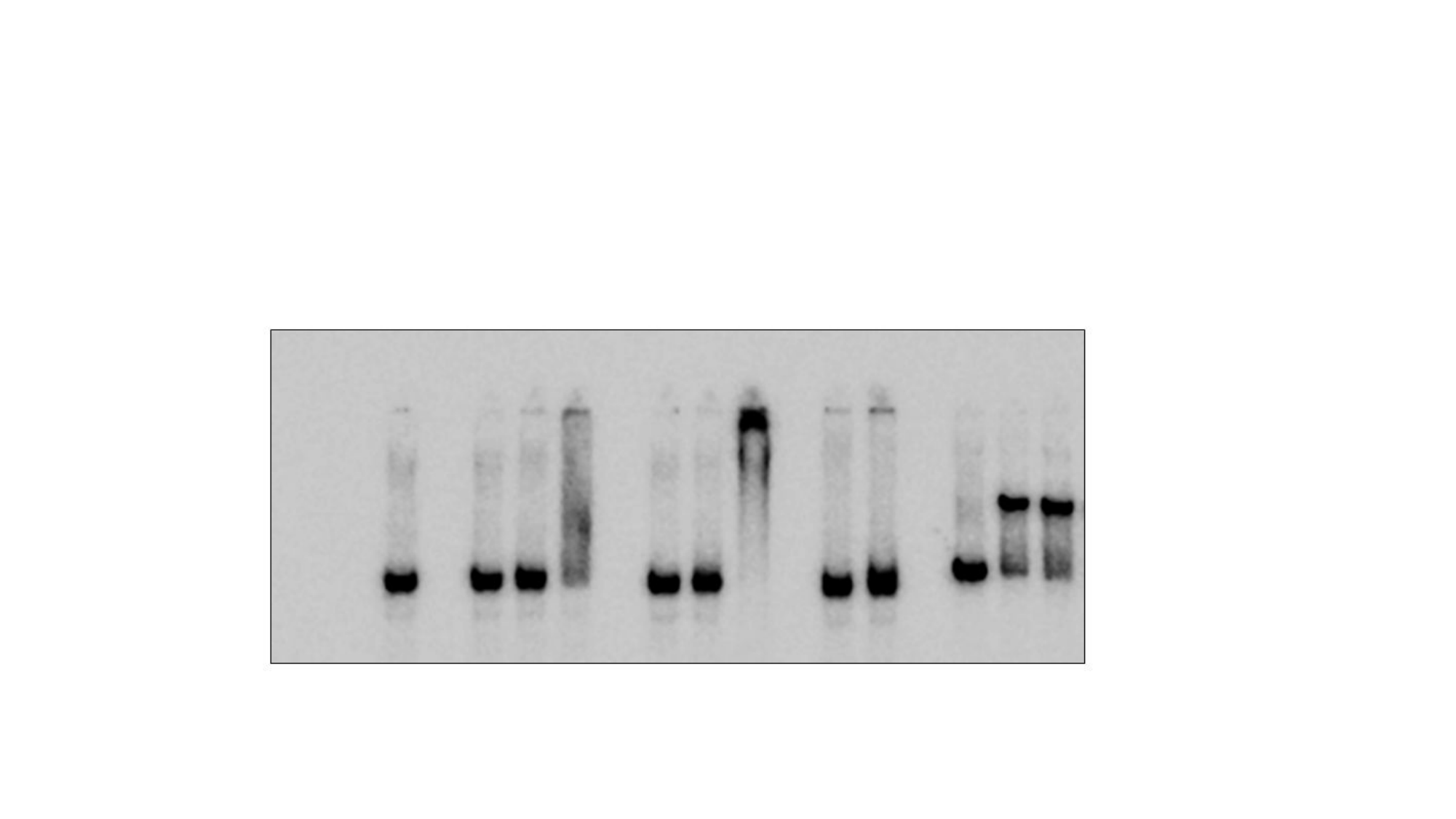

Supplement: Figure 2—figure supplement 1—source data 1. [file elife-92136-fig2-figsupp1-data1.pptx]

## Slide 1
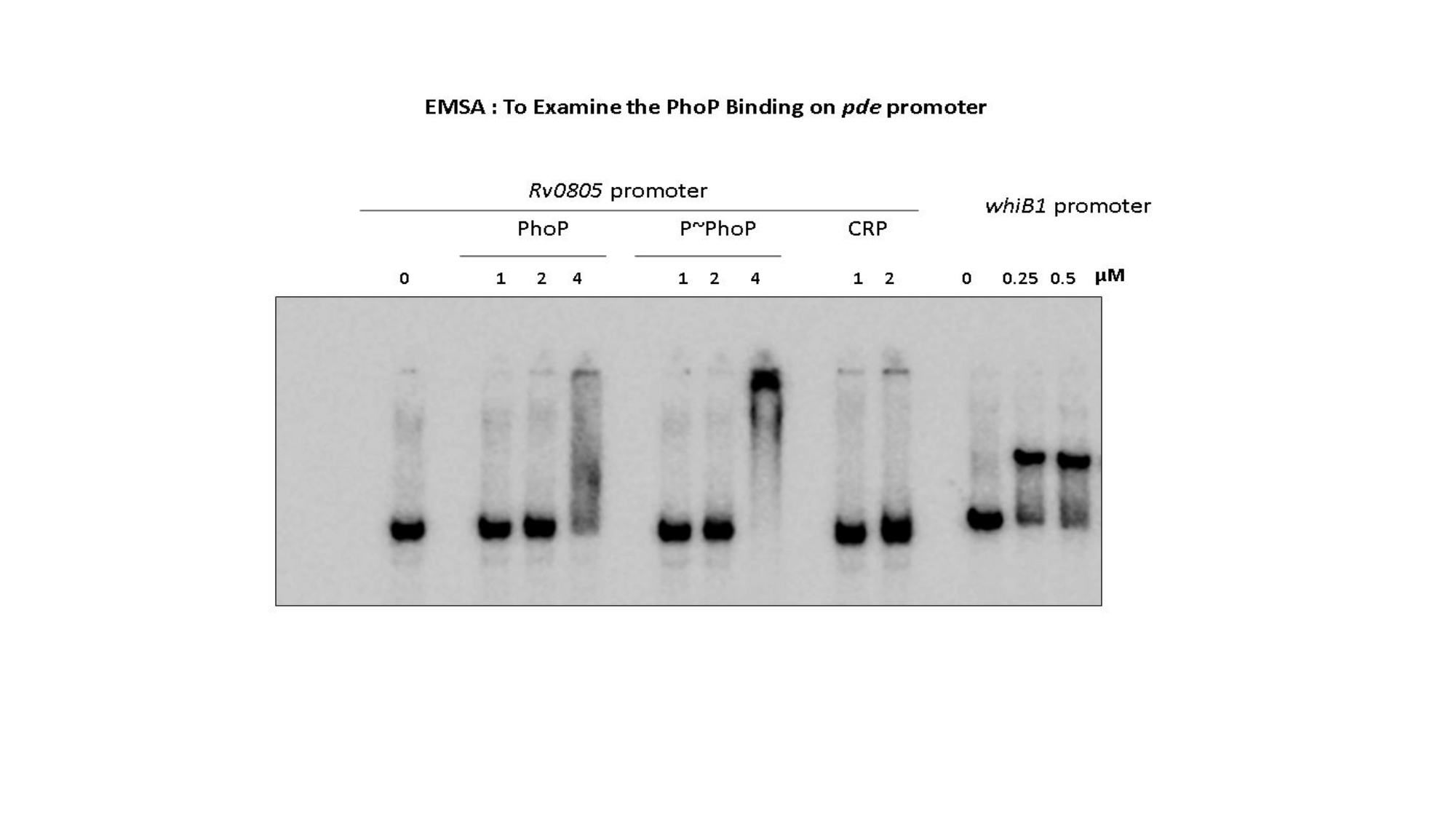

Supplement: Figure 2—figure supplement 1—source data 2. [file elife-92136-fig2-figsupp1-data2.pptx]

## Slide 1
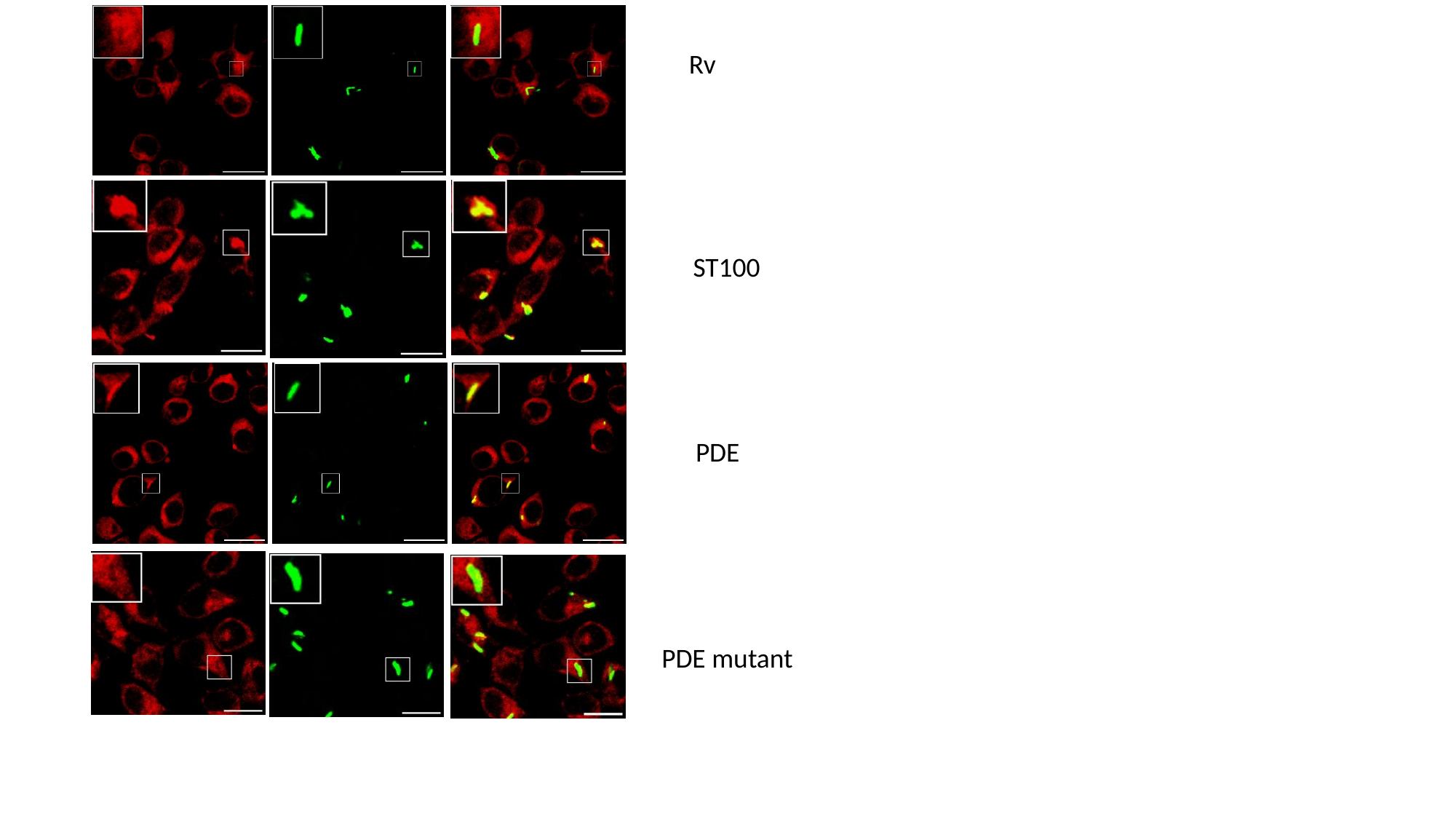

Rv
ST100
PDE
PDE mutant

Supplement: Figure 4—source data 4. [file elife-92136-fig4-data4.pptx]

## Slide 1
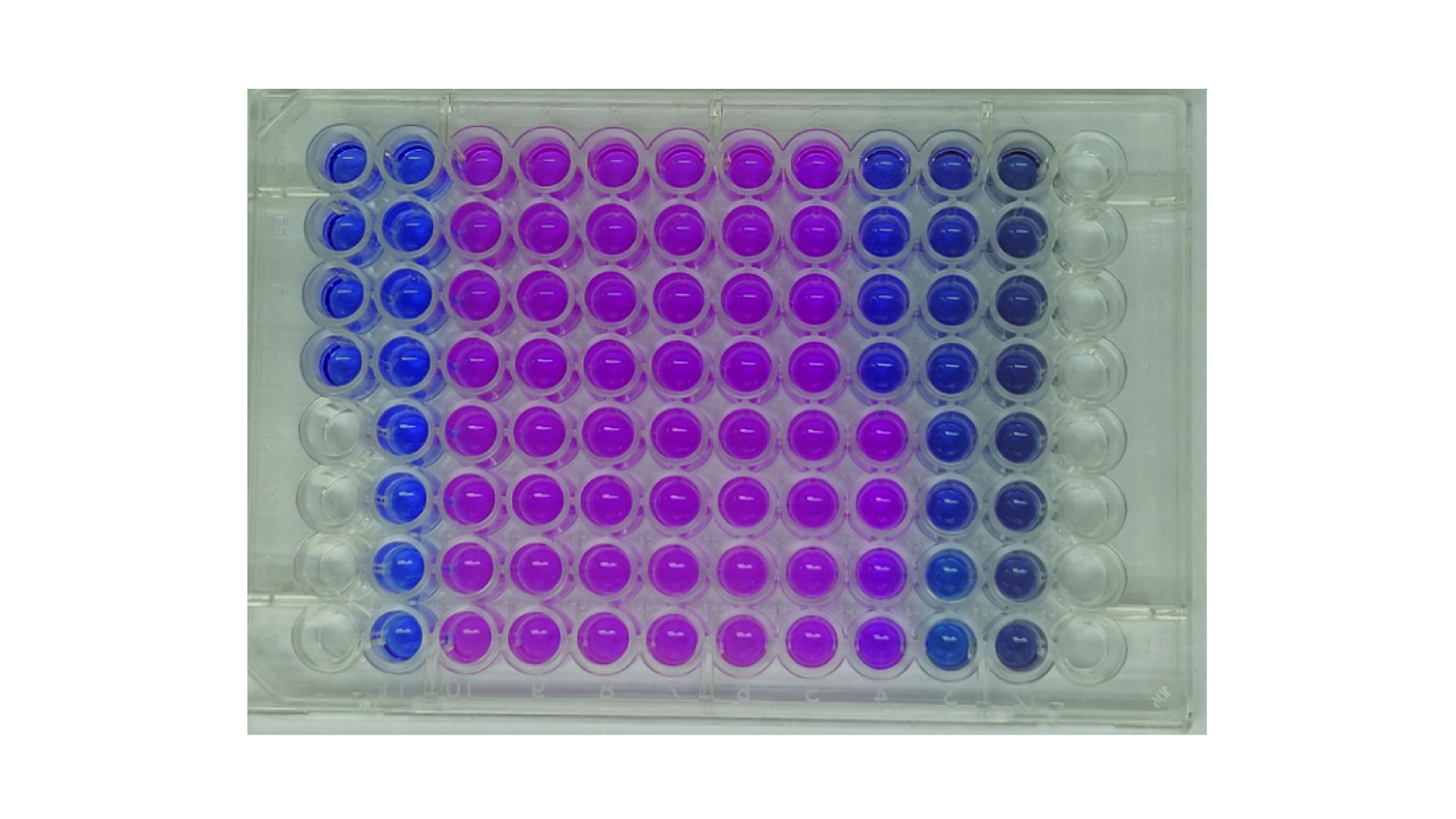

Supplement: Figure 4—figure supplement 1—source data 1. [file elife-92136-fig4-figsupp1-data1.pptx]

## Slide 1
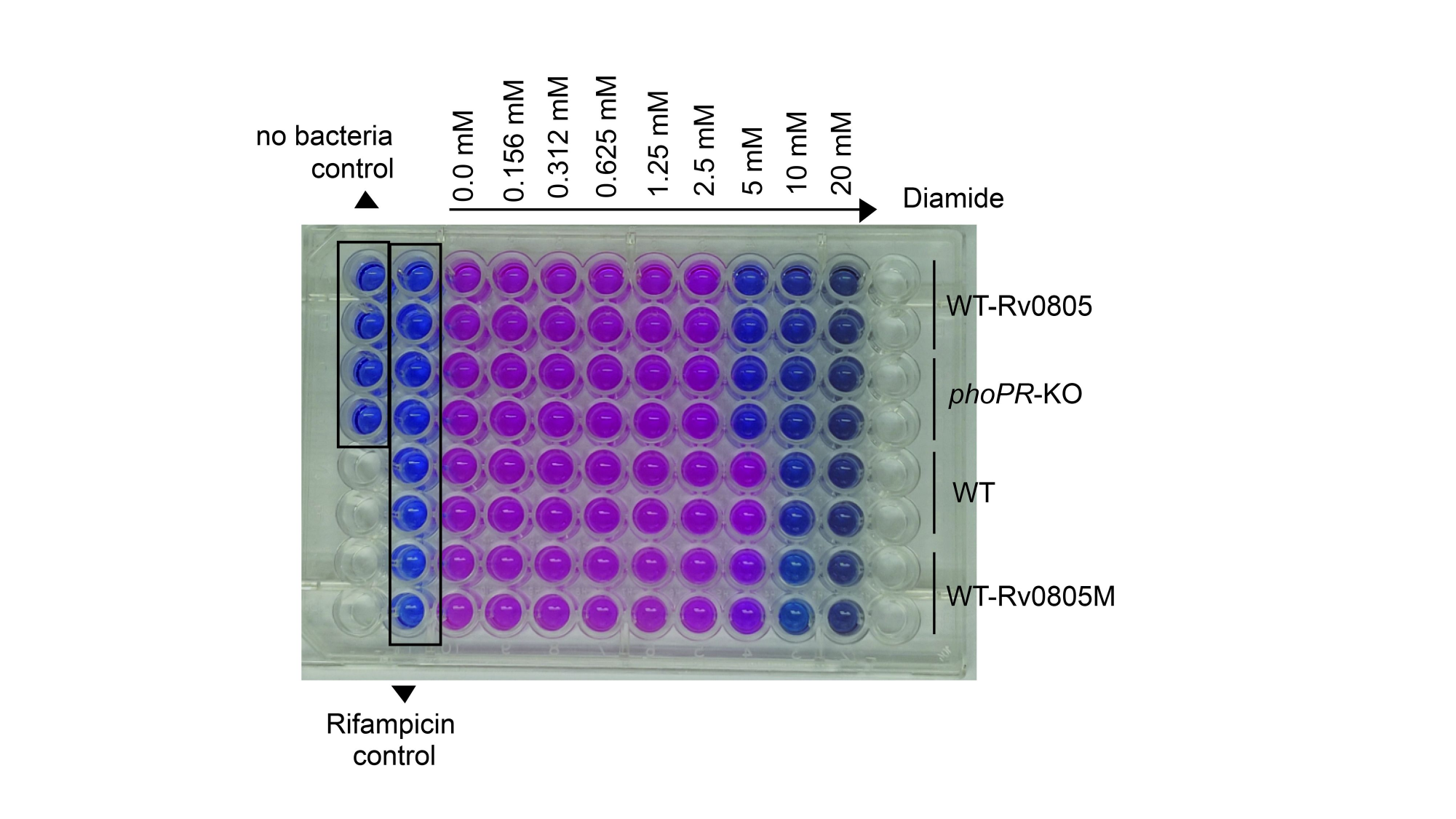

Supplement: Figure 4—figure supplement 1—source data 2. [file elife-92136-fig4-figsupp1-data2.pptx]

## Slide 1
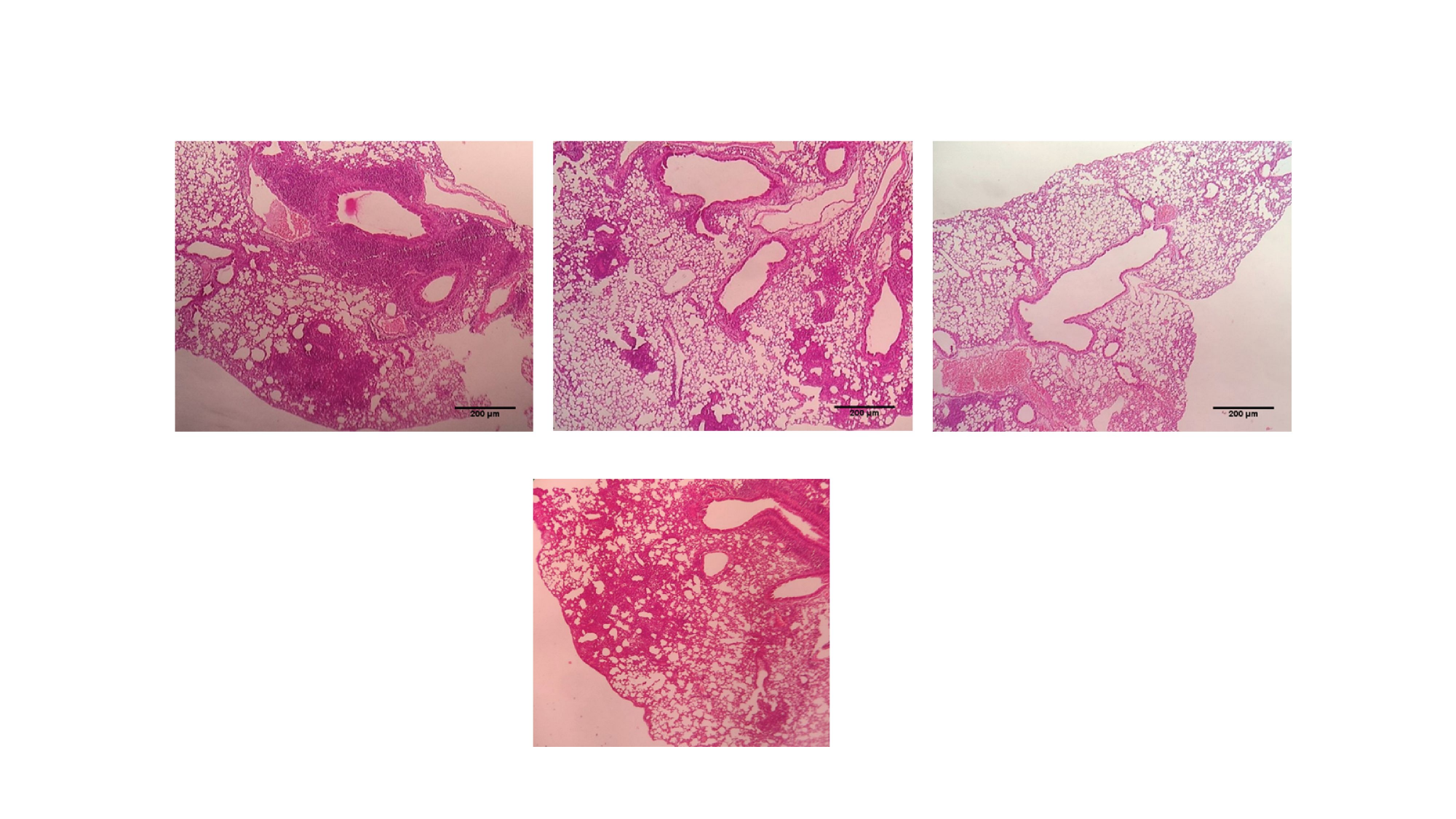

Supplement: Figure 5—source data 3. [file elife-92136-fig5-data3.pptx]

## Slide 1
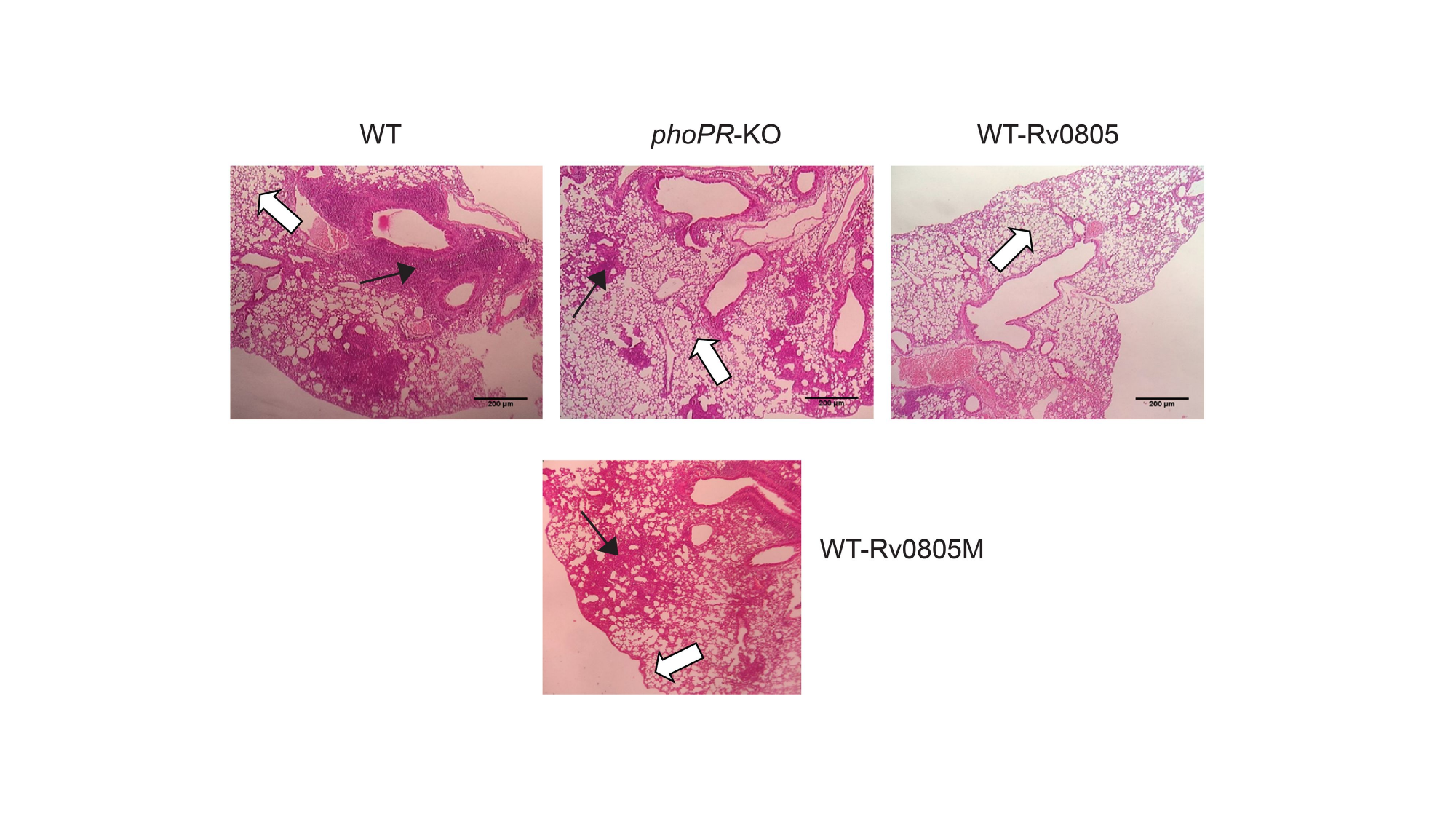

Supplement: Figure 5—source data 4. [file elife-92136-fig5-data4.pptx]
