# Supplementary material for: Mycobacterium tuberculosis PhoP integrates stress response to intracellular survival by regulating cAMP level
Source: eLife. 2024 May 13;13:RP92136. doi: 10.7554/eLife.92136 (PMC11090507; doi:10.7554/eLife.92136)
Supplement: Supplementary file 1. — (a) Sequences of oligonucleotide primers used in RT-qPCR and ChIP-qPCR measurements reported in this study. (b) Sequences of oligonucleotide primers used for amplification and cloning, and plasmids used in this study. [file elife-92136-supp1.docx]

SUPPLEMENTAL INFORMATION

*Mycobacterium tuberculosis* PhoP integrates stress response to intracellular survival by regulating cAMP level

Hina Khan^1,2^, Partha Paul^1,3^, Harsh Goar ^1,3,4^, Bhanwar Bamniya^1,3,5^, Navin Baid^1,6^,

Dibyendu Sarkar^1,5,^ *

^1^CSIR-Institute of Microbial Technology, Sector 39 A, Chandigarh 160036, India

^2^Present Address: Department of Physiology, Michigan State University,

Interdisciplinary Science and Technology Building, Lansing, MI 48824

^3^These authors contributed equally to this work.

^4^Present Address: Department of Medicine, Division of Hematology-Oncology

UT Southwestern Medical Center, Dallas, TX 75235

^5^Academy of Scientific and Innovative Research (AcSIR), Ghaziabad, India

^6^Present address: Department of Biosciences and Bioengineering

Indian Institute of Technology Bombay, Mumbai 400076, India

Running title: Regulation of mycobacterial cAMP

Key Words: cAMP level; Mycobacterium; PhoP; stress response; virulence regulation

*Address correspondence to: Dibyendu Sarkar, CSIR-Institute of Microbial Technology, Chandigarh, India

Tel.: 091-172-2880258; Fax: 091-172-2690585; E-mail: [dibyendu@imtech.res.in](mailto:dibyendu@imtech.res.in)

**Supplemental Table 1a**

Sequences of oligonucleotide primers used in RT-qPCR and ChIP-qPCR measurements

reported in this study

| **Primers^a^** | **Sequences (5’-3’)** | **Reference** |
| --- | --- | --- |
| FPrv0386RT | GGCCCAGATCCTTACCTTTC | This study |
| RPrv0386RT | TGTGCAGCACTTCCTGAGAC | This study |
| FPrv0891cRT | TCAAACGGTACGAGGGTGAT | This study |
| RPrv0891cRT | CACAACTGCATGACCCATTC | This study |
| FPrv1264RT | CAGCTAGGCGAAGTGGTGTC | This study |
| RPrv1264RT | GGGAAAGTTGTTGTCGGTGT | This study |
| FPrv1339RT | CGCCGTTGGTTATCGACTTC | This study |
| RPrv1339RT | AACAGTCGTCAATCTCCCCA | This study |
| FPrv1625cRT | TGAATTTGCCCCACCGAATC | This study |
| RPrv1625cRT | CAGCGCAATTGAAGGATCCA | This study |
| FPrv1647RT | GCCCAAGATGCTGTGAAGTC | This study |
| RPrv1647RT | AACTCACTTTGCGGGATCAG | This study |
| FPrv2488cRT | TTGCTGTTGGCATCATGTCT | This study |
| RPrv2488cRT | CTTCTGGGCATCATCTAGGC | This study |
| FPrv0805RT | GCCGAACTACGCAAATTCTT | This study |
| RPrv0805RT | ATCCAAAACACTCGGAATCG | This study |
| FPrv1357cRT | TCCTCGTCTACCAGCCAATC | This study |
| RPrv1357cRT | GAGACGTTGACGCTGACAAA | This study |
| FPrv2837cRT | AGCAGGACCTTGATGGACAG | This study |
| RPrv2837cRT | GTTCGACCTCCTTGAACACC | This study |
| FPphoPRT | GCCTCAAGTTCCAGGGCTTT | ([Khan *et al*, 2022](#_ENREF_6)) |
| RPphoPRT | CCGGGCCCGATCCA | ([Khan *et al.*, 2022](#_ENREF_6)) |
| FPpks2RT | GTTGTGGAAGGCGTTGTTAC | ([Goyal *et al*, 2011](#_ENREF_4)) |
| RPpks2RT | GTCGTAGAACTCGTCGCAAT | ([Goyal *et al.*, 2011](#_ENREF_4)) |
| FPmsl3RT | GTGAAAACAAACTTCGGTCAC | ([Goyal *et al.*, 2011](#_ENREF_4)) |
| RPmsl3RT | ACAAAGAGTTCAGTGTCAATCTCAG | ([Goyal *et al.*, 2011](#_ENREF_4)) |
| FPlipFRT | TAGTGGCCATCTCTCCGTTG | ([Bansal *et al*, 2017](#_ENREF_2)) |
| RPlipFRT | AGCGGCTCATAGAGGTCTTC | ([Bansal *et al.*, 2017](#_ENREF_2)) |
| FP16SrDNART | CTGAGATACGGCCCAGACTC | ([Khan *et al.*, 2022](#_ENREF_6)) |
| RP16SrDNART | CGTCGATGGTGAAAGAGGTT | ([Khan *et al.*, 2022](#_ENREF_6)) |
| FPrv0805up | CGGCGTTCTGGTATCTCG | This study |
| RPrv0805up | TAAGAGAACGTAATCCGG | This study |

^a^FP, forward primer; RP, reverse primer

**Supplemental Table 1b**

Sequences of oligonucleotide primers for amplification and cloning, and plasmids used in this study

| **Primers^a^** | **Sequences (5’-3’)** | **Reference** |
| --- | --- | --- |
| FPrv0805start | AATAATGATATCGTGCATAGACTT | This study |
| RPrv0805stop | AATAATAAGCTTTCAGTCGACGGGA | This study |
| FPrv0805N97A | TGGGTGATGGGTGCACACGACGACCG | This study |
| RPrv0805N97A | CGGTCGTCGTGTGCACCCATCACCCA | This study |
| 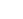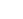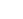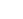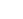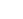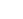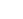FPphoPsg | GGGAGATCCAGCGCCTGTGCCCCG | This Study |
| 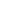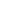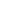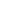RPphoPsg | AAACCGGGGCACAGGCGCTGGATC | This Study |
| FPrv0805sg | GGGAGCTCGACCAGGCCTCGGAGC | This Study |
| 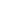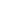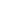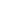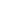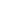RPrv0805sg | AAACGCTCCGAGGCCTGGTCGAGC | This Study |
| FPpRH2521seq | AAACTCTAGAAATATTGGATCG | This study |
| RPpRH2521seq | CCTAATGACCATGGTGACCTC | This study |
| **Plasmids** |  |  |
| p19Kpro^b^ | Mycobacteria expression vector, Hyg^r^ | ([De Smet *et al*, 1999](#_ENREF_3)) |
| p19Kpro-*phoP* | His_6_-tagged PhoP residues 1-247 cloned in p19Kpro | ([Anil Kumar *et al*, 2016](#_ENREF_1)) |
| p19Kpro-*phoP*(FLAG) | FLAG-tagged PhoP residues1-247 cloned in p19Kpro | This study |
| pSTKi^c^ | Integrative mycobacterial expression vector, Kan^r^*^c^* | ([Parikh *et al*, 2013](#_ENREF_7)) |
| pSTKi-*pde* | PDE (*rv0805*) residues 1-957 cloned in pSTKi | This study |
| pSTKi-*pde^M^* | PDE (*rv0805M*) Asn-97 codon mutated to Ala in pSTKi-*pdeM* | This study |
| pRH2502 | Integrative mycobacterial expression vector, Kan^r, c^ | ([Singh *et al*, 2016](#_ENREF_8)) |
| pRH2521 | Episomal expression vector, Hyg^r,b^ | ([Singh *et al.*, 2016](#_ENREF_8)) |
| pRH2521-phoPsg | pRH2521 vector expressing *phoP* guide RNA, Hyg^r,b^ | This study |
| pRH2521-rv0805sg | pRH2521 vector expressing *rv0805* guide RNA, Hyg^r,b^ | This study |

^a^FP, forward primer; RP, reverse primer;

^b^ hygromycin resistance (Hyg^r^)

^c^ kanamycin resistance (Kan^r^)

**References**

Anil Kumar V, Goyal R, Bansal R, Singh N, Sevalkar RR, Kumar A, Sarkar D (2016) EspR-dependent ESAT-6 Protein Secretion of Mycobacterium tuberculosis Requires the Presence of Virulence Regulator PhoP. *The Journal of biological chemistry* 291: 19018-19030

Bansal R, Anil Kumar V, Sevalkar RR, Singh PR, Sarkar D (2017) Mycobacterium tuberculosis virulence-regulator PhoP interacts with alternative sigma factor SigE during acid-stress response. *Molecular microbiology* 104: 400-411

De Smet KA, Kempsell KE, Gallagher A, Duncan K, Young DB (1999) Alteration of a single amino acid residue reverses fosfomycin resistance of recombinant MurA from Mycobacterium tuberculosis. *Microbiology* 145 ( Pt 11): 3177-3184

Goyal R, Das AK, Singh R, Singh PK, Korpole S, Sarkar D (2011) Phosphorylation of PhoP protein plays direct regulatory role in lipid biosynthesis of Mycobacterium tuberculosis. *J Biol Chem* 286: 45197-45208

Khan H, Paul P, Sevalkar RR, Kachhap S, Singh B, Sarkar D (2022) Convergence of two global regulators to coordinate expression of essential virulence determinants of Mycobacterium tuberculosis. *Elife* 11

Parikh A, Kumar D, Chawla Y, Kurthkoti K, Khan S, Varshney U, Nandicoori VK (2013) Development of a new generation of vectors for gene expression, gene replacement, and protein-protein interaction studies in mycobacteria. *Applied and environmental microbiology* 79: 1718-1729

Singh AK, Carette X, Potluri LP, Sharp JD, Xu R, Prisic S, Husson RN (2016) Investigating essential gene function in Mycobacterium tuberculosis using an efficient CRISPR interference system. *Nucleic Acids Res* 44: e143
